# Label-Free Electrochemical Biosensors for the Determination of *Flaviviruses*: Dengue, Zika, and Japanese Encephalitis

**DOI:** 10.3390/s20164600

**Published:** 2020-08-16

**Authors:** Ekaterina Khristunova, Elena Dorozhko, Elena Korotkova, Bohumil Kratochvil, Vlastimil Vyskocil, Jiri Barek

**Affiliations:** 1 School of Earth Sciences and Engineering, Department of Chemical Engineering, National Research Tomsk Polytechnic University, Lenin Avenue 30, 634050 Tomsk, Russia; yekaterinakhristunova@gmail.com (E.K.); elena-dorozhko@yandex.ru (E.D.); eikor@mail.ru (E.K.); bohumil.kratochvil@vscht.cz (B.K.); 2 UNESCO Laboratory of Environmental Electrochemistry, Department of Analytical Chemistry, Faculty of Science, Charles University, Albertov 6, 12843 Prague 2, Czech Republic; vlastimil.vyskocil@natur.cuni.cz; 3 Department of Solid State Chemistry, University of Chemistry and Technology, Prague, Technicka 5, 16628 Prague 6, Czech Republic

**Keywords:** label-free, electrochemical biosensors, *Flavivirus*

## Abstract

A highly effective way to improve prognosis of viral infectious diseases and to determine the outcome of infection is early, fast, simple, and efficient diagnosis of viral pathogens in biological fluids. Among a wide range of viral pathogens, *Flaviviruses* attract a special attention. *Flavivirus* genus includes more than 70 viruses, the most familiar being dengue virus (DENV), Zika virus (ZIKV), and Japanese encephalitis virus (JEV). Haemorrhagic and encephalitis diseases are the most common severe consequences of flaviviral infection. Currently, increasing attention is being paid to the development of electrochemical immunological methods for the determination of *Flaviviruses*. This review critically compares and evaluates recent research progress in electrochemical biosensing of DENV, ZIKV, and JEV without labelling. Specific attention is paid to comparison of detection strategies, electrode materials, and analytical characteristics. The potential of so far developed biosensors is discussed together with an outlook for further development in this field.

## 1. Introduction

The *Flavivirus* genus related to the family *Flaviviridae* includes more than 70 different viruses [1]. The genetic material of flaviviruses is represented by single-stranded RNA. Flavivirus is transmitted to people through the bites of infected ticks or mosquitoes [2]. Humans are usually considered as the dead-end hosts of these flaviviruses. The most frequent flavivirus infections from mosquitoes are caused by dengue virus (DENV), Zika virus (ZIKV), and Japanese encephalitis virus (JEV) [3]. This is probably the reason why most label-free biosensors introduced so far were focused on these flaviviruses, and the reason for their selection into this review. Importantly, there are significant variations in epidemiology or clinical aspects between these viruses. Nevertheless, all pathogens are closely related genetically; the variation of amino acid sequences between individual subtypes is 5–6%. Therefore, in countries where a variety of different flaviviruses co-exist, cross-reactivity between them may occur, and accurate diagnosis become a challenging task [4]. Infection with any flavivirus subtype causes serious damage to human health. Haemorrhagic and encephalitis diseases are the most common severe consequences of flaviviral infection [5]. The immunization of people is the most effective protection against the serious consequences of these diseases, but despite the availability of vaccines [6,7,8], the number of cases of flaviviral infection have been increasing over the past two decades [9]. The growth in the incidence rate is the result of a complex interconnection of socio-economic factors [10] and of the fact that vaccine coverage is insufficient for many risk groups. Other significant factors in the growth of flaviviral diseases are global climate change [11], deforestation [12], population growth [13], and even mutations in the sequence during pre- to post-epidemic transition (alanine-to-valine, in ZIKV-NS1 protein) [14], which lead to an increase of viruses epidemic transmission.

After flaviviral infection, specific viral proteins, viral nucleic acids, intact viral particles, and antibodies are generated as an immune response [15]. Among them, non-structural proteins (NS1–NS5) are important molecules in immunosensing and molecular research for the diagnostic of flaviviruses [16]. It is interesting that in the acute phase of Dengue infection, NS1 concentration has been estimated in 0.04–2 µg/mL for primary infection, but only 0.01–2 µg/mL for secondary infection [17]. Therefore, biosensors can be used to discriminate between primary and secondary infection [18]. Nowadays, reverse transcriptase polymerase chain reaction (RT-PCR), enzyme-linked immunosorbent assay (ELISA), and plaque reduction neutralization test (PRNT) are the most common methods for detection of these specific analytes [19,20,21]. However, traditional techniques require relatively expensive equipment, and often are not suitable for rapid on-site analysis because of limited resources [22,23]. For instance, RT-PCR technique requires performing steps before polymerase chain reaction (PCR), which basically includes the reverse transcription, creating DNA from viral RNA [24]. The development of progressive diagnostic tools applicable for the determination of specific pathogenic flaviviruses in biological fluids is a very promising direction. In the last few decades, the researchers have shown particular interest in the development of electrochemical biosensors for the diagnosis of DENV, ZIKV, and JEV [25,26,27]. Electrochemical biosensors measure the intensity of the electrical signal provided by a special interaction with a target analyte and proportional to its concentration. Electrochemical biosensors are of special interest due to their high sensitivity, selectivity, relatively low equipment acquisition costs, and suitability for miniaturization. Considering these circumstances, several scientific groups have developed new electrochemical biosensor systems for the quantification of flaviviruses [28,29,30,31,32,33,34,35,36].

Label-free biosensors represent a special class of electrochemical biosensors for the determination of flaviviruses [37,38,39], where the quantification of analytes of interest is based on techniques such as electrochemical impedance spectroscopy (EIS), voltammetry, or amperometry without the need of any signal label (e.g., enzymes, metal nanoparticles, etc.). The presence of the label can affect the kinetics and the specific binding of analytes resulting in a systematic error in the measurement [40]. Direct detection eliminates the marking steps, thus reducing the time and cost of analysis. Label-free biosensors discover new horizons in designing portable lab-on-a-chip biosensors for detection of different pathogens. Different types of recognition elements and electrode materials are used for developing such highly sensitive label-free sensors, which are highly specific for the detection of target molecule and determine the possibilities and limitations of the biosensor used.

In this review, we have focused on recent advanced applications of electrochemical label-free biosensors in diagnostics of flaviviruses. Specific attention is paid to comparison of detection strategies, electrode materials, and analytical characteristics. The potential of so far developed biosensors is overviewed together with an outlook for further development in this field. The article is basically organized by different flaviviruses and, in the chapters devoted to individual flaviviruses, sensing principles were used as second classification criterion.

## 2. Electrochemical Detection Methods

Label-free electrochemical biosensors transform information related to electrochemical and specific biochemical reactions into an appropriate signal, which can be used both for qualitative and quantitative purposes [41]. Different electrochemical detection methods were designed and applied to convert the electric signal into analytically useful information related to flaviviral infection: potentiometry (measuring the potential of an indicator electrode) [42,43], conductometry (measuring the conductivity or resistance) [44], amperometry/voltammetry (current measurement as a function of imposed potential) [28,45,46,47], and EIS (measuring the impedance of a system) [48,49,50]. The majority of publications over the past decades focus on voltammetric and EIS techniques to obtain label-free biosensors with a high sensitivity and, therefore, low limit of detection (LOD), which are the main figures of merit of biosensors. The sensitivity is defined as the slope of the analytical calibration curve, and an analytical method is sensitive when a small change in analyte concentration causes a large change in the response [51]. The LOD is the concentration or the quantity derived from the smallest signal that can be detected with acceptable degree of certainty for a given analytical procedure [51,52]. Commonly, the limit of detection is calculated as LOD = 3*s*/*b* (where *s* is the standard deviation of the signal of blank sample, and *b* is the slope of the straight section of the calibration curve) [53]. Another variant for LOD estimation is calculation from the signal-to-noise ratio (*S/N* = 3) [54]. In any case, the choice of analytical method can significantly affect the measurement result.

### 2.1. Electrochemical Impedance Spectroscopy and Conductometry

**Figure 1:**
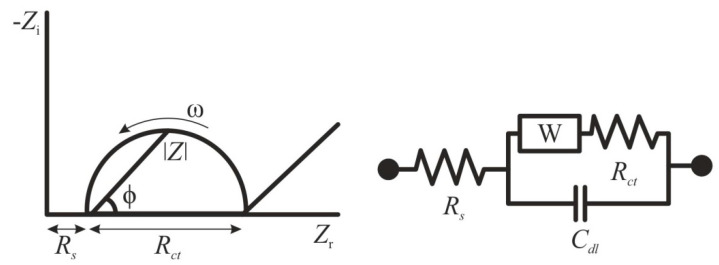
Randles equivalent circuit of an electrochemical cell. *Z*_i_—imaginary impedance, *Z*_r_—real impedance, *R*_s_—solution resistance, *R*_ct_—charge transfer resistance, *W*—Warburg impedance, *C*_dl—_double-layer capacitance, *ϕ*—phase angle, *ω*—angular frequency.

In recent years, many researchers have applied EIS for the determination of specific pathogenic flaviviruses [55,56,57]. This method stands out among other electrochemical methods, since the analysis proceeds without irreversible changes in the electrode material, and does not require a high consumption of chemical reagents [58]. EIS is based on the detection of changes in resistance on electrode before and after modification by a biological material [48]. The impedance *Z* of a system is generally measured by applying a voltage perturbation with a small amplitude and detecting the current response. The impedance is a complex value, since the current can differ not only in terms of the amplitude, but it can also show a phase shift *ϕ* compared to the voltage–time function. Thus, the value can be described either by the modulus |*Z*| and the phase shift *φ* or alternatively by the real part *Z*_r_ and the imaginary part *Z*_i_ of the impedance (Figure 1) [58]. The registration of the EIS spectra is carried out in the presence of a redox probe—a substance capable of rapid reversible single-electron redox transformation [59]. Ferri/ferrocyanide systems or ruthenium complexes are usually used as such redox markers [60,61]. Typically, the obtained impedance spectra are fitted to an equivalent circuit using a Nyquist plot for illustration, and the change in charge transfer resistance is correlated to the target biological material concentration [62]. For the situation of an electrode in contact with an electrolyte, the so-called Randles circuit is used (Figure 1), comprising the double-layer capacitance *C*_dl,_ the solution resistance *R*_s_, the charge transfer resistance *R*_ct_, and the Warburg impedance *W.*

By taking measurements with surface-modified sensing electrodes, the presence of redox probe results in a well-defined charge transfer resistance *R*_ct_. If the redox probe is omitted or a blocking layer is applied to the electrode, rather capacitive impedance behavior will be detected (since *R*_ct_ will become extremely large). Therefore, a binding effect at the electrode can be observed by following the change in *R*_ct_ [58]. EIS is characterized by extremely high detection sensitivity of analytes (LOD reaches subnanomolar concentrations); however, the accuracy of determination highly depends on the quality of the electrode surface preparation. Moreover, EIS requires controlling false positive analyses which are associated with the dynamics of the sensing interface and have inherently low signal-to-noise ratio. Control of the stability of the signal-to-noise ratio, which controls the dynamics of the sensing interface, should be the first planned requirement rather than the detection of signal changes on the electrode surface [63].

In addition to EIS, conductometry was used as successful example for different viruses detection [44]. Conductometry determines the interactions between target and capture by calculating the conductance of various ionic spices [64]. The intrinsic negative charge of biomolecules can have a large effect on both ionic and electronic conductivity of a system and has been studied to develop label-free biosensors by measuring the electrical signal of the system [44]. For instance, field-effect transistor (FET) uses the system electronic conductivity for the determination of biomolecules where the current passing between a source and a drain is controlled by the potential connected to a gate [65].

### 2.2. Voltammetry, Amperometry

Cyclic voltammetry (CV), differential pulse voltammetry (DPV), and square-wave voltammetry (SWV) are most popular voltammetric techniques that allow for the determination of low concentrations of flaviviruses, which is attractive for biomedical and biological research [66,67,68]. These methods are successfully used in the creation of label-free biosensors for the determination of viral analytes. Various mechanisms of the redox reaction, the state of the microenvironment of the double electric layer in biosensors, the estimation of the constants of binding of the analyte to the receptor layer on the electrode, and the diffusion limitations of biological analytes can be estimated using these methods. The majority of publications are devoted to registration of the resultant current of the redox probe corresponding to changing electrode potential. Ferri/ferrocyanide, ferrocene, ferrocenethanol systems, and ruthenium complexes are the most frequently used redox probes [50,69,70]. The electrochemical behavior of redox probes depends on the receptor layer and the biosensor operating principle. In most studies, when a complex is formed between the molecules of the receptor layer and the analyte target, the recorded current of the redox probe decreases.

In addition to voltammetry, amperometry is one of the important techniques that was used for developing biosensors [45,71,72,73]. The amperometric response is mainly based on the reduction–oxidation of ferri/ferrocyanide probe on the electrode surface proportional to the concentration of target analyte in the sample.

It can be summarized that the main advantage of voltammetric techniques is easily available instrumentation, low running and investment costs, and user friendliness. They are most frequently used in practical laboratories, which makes them most popular in flaviviruses monitoring. The main advantage of amperometry is its compatibility with measurements in flowing system, resulting in shorter times of analysis and increased productivity with comparable sensitivity and LOD.

## 3. Electrochemical Biosensors for DENV Diagnostic

Label-free electrochemical biosensors, in which the mechanism of signal transduction is based on the measurement of current as a result of oxidation/reduction of a probe or a modulation of electrochemical impedance on electrode surface during bioconjugation process, offer sensitive and rapid way to diagnose DENV. Depending on the nature of the bio-recognition components on the receptor layer of label-free electrochemical biosensors, three groups can be distinguished for determining DENV: (i) DNA/RNA biosensors, (ii) immunosensors, (iii) peptide-based biosensors. There are four different antigenic compositions of DENV serotypes: DENV1, DENV2, DENV3, and DENV4 [74]. DENV virion consists of three structural proteins plus the lipoprotein membrane and seven non-structural proteins. One of non-structural dengue proteins NS1 has diagnostic and pathological significance, because it is prevalent in all four serotypes of DENV [75]. Infection with any one serotype provides lifelong immunity to this specific serotype; however, cross-immunity to other serotypes lasts only a few months. Significant genetic variation occurs in each serotype of the virus, as a result of which phylogenetically different genotypes are formed [76]. It is relevant to create the system capable to detect all DENV serotypes. Therefore, specific attention in the development of biosensors was paid to the design and surface properties of the electrodes that are used for immobilization of various biological molecules. The electrode surface should have a large electroactive area, maintain a high activity of immobilized biomolecules, have a uniform distribution of active binding sites over the entire electrode area, have physical and chemical resistance of the coating in contact with liquid, and be capable of repeated use of the surface.

In developing label-free biosensors for complex matrix samples (e.g., blood, serum), important issue is the fouling of the working electrode resulting in the deceased signal [77]. Researchers paid attention to overcome this problem for flaviviruses detection, for example, by the designing of disposable chips [78,79]. Further advantages of this approach are connected with the fact that, in outbreaks of infections, the importance of using affordable and easy-to-handle devices is essential in order to avoid cross-contamination and, thus, false positive results.

### 3.1. DNA and RNA Biosensors for DENV Diagnostic

Some of DNA/RNA biosensors are based on the phenomenon of hybridization with oligonucleotides of various DENV serotypes. The two strategies are used. In the first case, hybridizing solution mixture is immobilized on the electrode surface and a direct electrochemical response from the hybrid duplex is recorded [66]. In the second case, a DNA probe is immobilized on the surface of the sensor, which is complementary to the target DNA in the analyzed solution, and electrochemical signal from redox/capture probes is recorded by voltammetric methods before and after hybridization [80].

**Figure 2:**
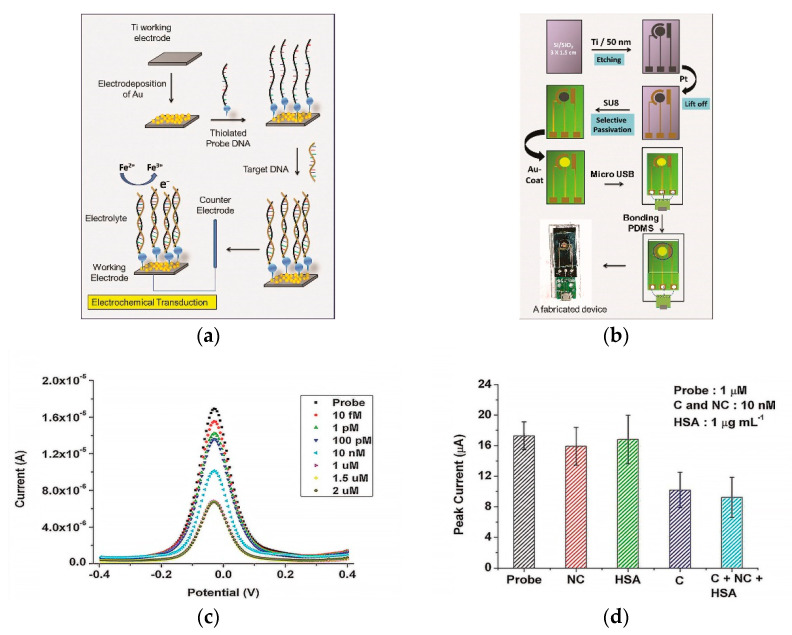
(**a**) Schematic representation of the gold nanostructure-based working electrode fabrication and the subsequent DNA immobilization protocol; (**b**) schematic representation of the process flow for the miniaturized electrochemical biosensor platform fabrication. SU8—Epoxy-based negative photoresist, PDMS—poly(dimethylsiloxane); (**c**) differential pulse voltammetry (DPV) recordings for Ti/Au/probe electrodes at different target DNA concentrations; (**d**) influence of interferents (NC—non-complementary DNA, C—complementary DNA, HSA—human serum albumin) as non-complementary targets at probe concentration 1 µM. Adapted with permission from [50].

The immobilization technique of a capture probe can influence biosensor efficiency. For instance, several studies are devoted to the development of DNA/RNA biosensor platforms modified with metals or their oxides to increase the electroactive surface of the sensor, which provides stable and sensitive electrochemical detection of the DENV of various serotypes [66,81]. The presence of metals or their oxides on the electrode surface contributes to the biocompatibility and target specificity with DNA molecules [82]. A successful example with nanostructured gold electrode (AuE) surfaces for DENV determination is the paper of Tripathy et al. [80]. The authors made a miniature electrochemical sensor as a promising point-of-care platform for determining the DENV specific consensus primer at the subfemtomolar concentration level (LOD 9.7 × 10^−16^ mol/L). The development stages of the platform included the production of nanostructured gold on a titanium working electrode by electrodeposition, gold nanoparticles (AuNPs) being excellent material for immobilization of thiolated DNA probes with the formation of self-assembled monolayers [83] of the receptor layer, for the hybridization of a complementary target DNA (Figure 2a). Moreover, the authors included micro-USB-based electrical interface on the miniaturized platform for presumptive diagnosis of DENV at the point of care (Figure 2b). Upon successful hybridization, the decreased oxidation current from a redox pair [Fe(CN)_6_]^3−/4−^ was registered by DPV (Figure 2c), including the influence of selected interferents (Figure 2d).

Electrospun semi-conducting manganese(III) oxide (Mn_2_O_3_) nanofibers were also used as substrate elements of electrochemical platforms for detecting DNA hybridization of the DENV [81]. Tripathy et al. developed a label-free biosensor for detection of a consensus DENV primer by using various electrochemical methods (CV, DPV, EIS) with a redox pair [Fe(CN)_6_]^3−/4−^ at the zeptomolar concentration level (LOD 1.2 × 10^−19^ mol/L). Another significant approach to improve the properties of the electrode surface for the sensitive determination of DENV is a biosensor based on nanoporous membrane-like structures. Rai et al. created a sensitive DNA biosensor based on a nanoporous alumina membrane constructed using 59-aminated DNA probes immobilized on the walls of an alumina channel [84]. This biosensor used [Fe(CN)_6_]^3−/4−^ to generate DPV sensing signal, with LOD of DENV1 ≈ 10^−5^ mol/L.

The intrinsic negative charge of DNA/RNA molecules can have a deep effect on both ionic and electronic conductivity of a system. This phenomenon has been explored to develop label-free biosensors by measuring the electrical signal of the system due to molecules hybridization [85]. One such example is the FET that uses the electronic conductivity of the system for the detection of DNA molecules [44]. Zhang et al. investigated platform based on peptide nucleic acid (PNA)–DNA hybridization for DENV detection on a n-type FET nanowire sensor [86]. Change in charge upon hybridization further induces an increase in resistance which constitutes the basis of this detector sensing mechanism. Percentage change in resistance was calculated using the following formula: [(resistance after hybridization−resistance before hybridization/resistance before hybridization)] × 100. This assay is very fast (30 min), with LOD of 1.0 × 10^−14^ mol/L.

Conductometry has also found applications for the detection of DENV, where conductance with an ion current signature was used for quantification of hybridized nucleic acids. For instance, Senapati et al. demonstrated a platform for detection of DENV based on an ionic diode feature of an anion exchange nanoporous membrane [44]. This biosensor gave LOD 1.0 × 10^−12^ mol/L and 15 min assay time in analysis of nucleic acid in real samples. Moreover, this platform allows to differentiate two serotypes DENV2 and DENV3.

### 3.2. Immunosensors for DENV Diagnostic

In the development of immunosensors, presence of antibodies plays a crucial role; they can act as detectable compounds or recognition molecules. Generally, antibodies as a recognition element immobilized on the surface of biosensor for the determination of DENV are used. For instance, Cheng et al. developed a sensitive membrane electrochemical immunosensor for the label-free detection of DENV2, where anti-DENV2 monoclonal antibody is used as the bio-recognition element [87]. The authors proposed a method for measuring electrode’s Faradaic current response to redox probe ferrocenethanol by DPV, which decreased as the result of the formation of immune complexes in alumina nanochannels between specific monoclonal antibodies and DENV2, with LOD of 1 pfu/mL (plaque-forming units per millilitre). Some other works following the same strategy (using nanoporous membranes) were published [35,54]. In both membrane immunosensors, the receptor layer of antibodies was immobilized within the thin alumina layer for identification of DENV, and these studies provided opportunity for DENV detection in clinical diagnosis by the EIS technique.

Several recent approaches have taken advantage of the functionalization with *anti*-DENV NS1 antibodies on the metal electrode surfaces. For example, Darwish et al. suggested to use indium tin oxide (ITO) electrode in combination with AuNPs [88]. Wasik et al. utilized a network of self-assembled single-walled carbon nanotubes (SWNT) on lithographically patterned interdigitated gold microelectrodes [89]. Cecchetto et al. employed a mixed self-assembled monolayer consisting of 11-mercaptoundecanoic acid (MUA) and 6-mercaptohexanol (6COH) prepared on a AuE [37]. All described immunosensors were developed for the label-free detection of the unstructured DENV protein NS1 in actual serum from patients infected with DENV by the EIS technique with LOD of 5 ng/mL [88], in adulterated artificial human saliva with LOD of 1 ng/mL [89], and in neat serum with LOD of 30 ng/mL [37]. In the same line, Cecchetto and coworkers recently proposed an original approach for detection of DENV protein NS1 on the surface of AuE modified with recognition element (*anti*-DENV NS1 antibody) attached to the ferrocene-tagged peptide structure [90]. The response of the interaction between antibody-antigen was monitored by EIS.

**Figure 3:**
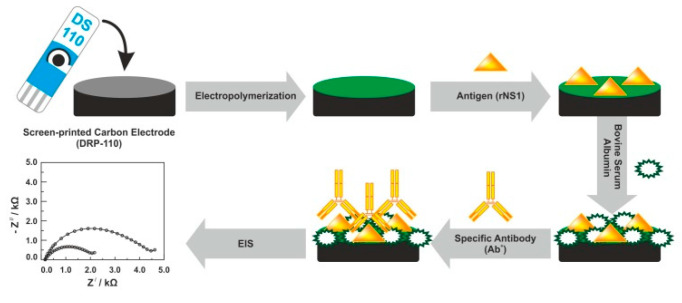
Fabrication protocol of the impedimetric immunosensor. DRP-110—carbon model of screen-printed electrodes, rNS1—non-structural recombinant protein 1, EIS—electrochemical impedance spectroscopy. Adapted with permission from [93].

In addition to antibodies, NS1 antigens were used as the receptor electrode layer of biosensors for label-free detection of DENV antibodies. For instance, Santos et al. demonstrated a new tool for detecting *anti*-DENV NS1 antibodies in real serum samples on a screen-printed carbon electrode (SPCE) by the EIS technique (Figure 3) [91], where results obtained by the electrochemical method were verified using a standard ELISA method. An original approach of particular interest is the recognition element (*anti*-NS1 antibody) attached to the ferrocene-tagged peptide structure using standard EDC (1-ethyl-3-(3-dimethylaminopropyl)carbodiimide)/NHS—(*N*-hydroxysuccinimide) protocol [92].

Another interesting approach, described by Santos et al., is based on the immobilization of PEG (poly(ethylene glycol)-thiol (with low fouling features) at the AuE surface for detecting of *anti*- DENV NS1 antibodies [51]. A low fouling component (PEG) was used in order to avoid non-specific interactions. In the same line, to prevent nonspecific binding (fouling), Darwish et al. modified the bare ITO electrode with 4-sulfophenyl, 4-trimethylammoniophenyl, and 1,4-phenylenediamine [88]. These antifouling molecules with charged terminal groups can counter the non-specific adsorption of the various proteins naturally found in human sera [94].

Recent trend in DENV detection methods using label-free biosensors is the application of molecularly-imprinted polymers (MIPs) [95,96]. MIP-based biosensors attract attention due to simplicity, easy mass production, improved shelf-life, and low costs of sensor. For instance, Arshad et al. developed the MIP-based biosensor for detection of DENV protein NS1 in real human serum samples on the surface of SPCE modified with electrically conductive polysulfone nanofibers and dopamine. In MIP sensing, the selective recognition of target analyte is based on its chemical as well as geometrical fitting into imprinted cavities of polymer matrix [97]. The self-polymerization of dopamine at room temperature helps to maintain the precise structure of template (NS1), which results in generating geometrically fit imprinted sites for further target analyte detection. A proposed MIP-based biosensor can selectively detect NS1 concentrations as low as 0.3 ng/mL.

The concept of FET has been also found application in the development of immunosensors for the detection of DENV [98]. Viera and coworkers proposed an original approach for the label-free recognition of DENV NS1 using the metal-oxide-semiconductor FET as a platform for detection of protein interactions [98]. The detection principle is based on the change in the charge distribution when a target DENV NS1 was recognized by immobilized anti-DENV NS1 antibodies on the FET surface. The authors noted that only the charge change due to antigen–antibody interactions that occurs within the Debye length can be measured by using a FET immunosensor [65]. Therefore, AuE was used as a material able to detect antigen–antibody interactions in FET immunosensors, due to exhibition of non-Nernstian behavior (pH sensitivity < 59.15 mV/pH). This immunosensor has LOD of DENV NS1 of 0.25 μg/mL.

### 3.3. Peptide-Based Biosensors for DENV Diagnostic

**Figure 4:**
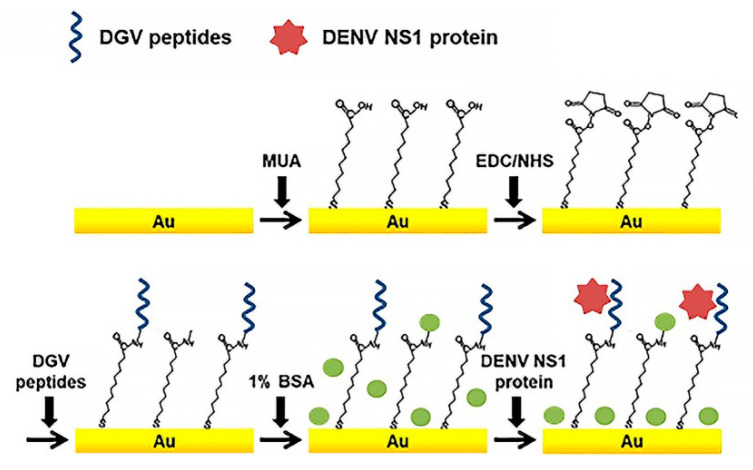
Schematic illustration of the peptide-decorated electrochemical sensor for the detection of dengue fever biomarker, NS1. DGV peptides—synthetic phage-displayed peptides, specific for NS1, MUA—11-mercaptoundecanoic acid, EDC—1-ethyl-3-(3-dimethylaminopropyl)carbodiimide, NHS—*N*-hydroxysuccinimide, BSA—bovine serum albumin. Adapted with permission from [44].

Just a few of DENV-specific peptide sensors have been reported [33,44]. Linearity, unique small size, cost efficiency, and biocompatibility of peptides makes them more perspective as a bio-recognition element than antibodies. Peptides can easily bind to the electrode surfaces of various materials, such as metal nanoparticles (NPs) or carbon nanotubes (CNTs), for bioassays. Recently, an effective label-free electrochemical immunosensors based on binding affinities of five synthetic affinity peptides for the detection of non-structural protein NS1 (Figure 4) were developed [68]. Relative binding affinities of these synthetic peptides were determined by SWV and EIS.

As noted in introduction section, NS1 is a specific and sensitive biomarker for diagnosis of DENV. Five synthetic peptides (DGV BP1–BP5) with different amino acid sequences were chemically synthesized for testing as candidates for affinity binding to SN1. For instance, to examine the binding affinity of a peptide with flexibility and a non-fouling nature, the DGV BP4 peptide was synthesized by incorporating the non-fouling peptide. The sensor performance for DENV NS1 determination was monitored by SWV and EIS using a redox pair [Fe(CN)_6_]^3−/4−^. DGV BP1 (EHDRMHAYYLTRGGGGSC) was selected as a promising recognition peptide, with LOD of SN1 ≈ 1.49 μg/mL. Lim and coauthors also used CV, SWV, and EIS for monitoring interaction between a peptide and DENV2 protein NS1 [55].

In summary, the technical approaches for implementing the DENV detection method determine the basic principle to differentiate these described DNA/RNA and peptide biosensors and immunosensors for the determination of DENV. Table 1 summarizes types of electrodes, methods, targets, and bio-recognition layers applied for DENV determination. As a result of different samples and approaches used, the units used for LOD are different and cannot be converted to the unified ones. The same holds for Table 2 and Table 3.

It can be concluded that present electrochemical biosensors showed practical applicability of detection of different DENV serotypes. However, none of the commercial rapid tests distinguishes the DENV serotypes [106]. The main advantage of using commercial test strips is the fact that it is possible to detect dengue virus using both dengue NS1 antigen and anti-dengue antibodies (IgM/IgG) with one device. The same holds for ZIKV commercial test strips (Table 2).

## 4. Electrochemical Biosensors for ZIKV Diagnostic

ZIKV is relatively new virus discovered in the middle of the 20th century [107]. Therefore, studies in the development of label-free electrochemical biosensors for its detection are limited. ZIKV can cause serious sequelae such as fetal microcephaly or Guillain-Barré syndrome [108]. Epidemiological monitoring of infection has significant role for the diagnosis of ZIKV. Considering that most of the cases of ZIKV infection are in countries with limited resources, there is an intensive search focused on the development of efficient, low-cost label-free biosensors for selective and rapid determination of ZIKV in real samples at epidemic areas.

For the determination of ZIKV, a wide range of bio-recognition components on the receptor layer of label-free electrochemical biosensors was so far used (Table 2). Specific attention is paid to the exclusion of cross-reactivity with other flaviviruses, in particular DENV. For instance, several studies are devoted to the development of biosensor steps aimed at eliminating cross-reactivity with DENV [109,110]. The biosensors used a non-structural protein 1 (NS1) as an important molecule for the selective detection of ZIKV. Studies have shown that NS1 has the potential for the selective diagnosing of ZIKV without cross-reactivity with another flaviviruses [111,112]. Faria et al. introduced a new platform based on ZnO nanostructures immobilized with ZIKV NS1 antibody on a printed circuit board (PCB) followed by CV evaluation [109]. Their findings suggest a high selectivity to ZIKV (LOD 1 pg/mL), without cross-reactivity with DENV. Another group has proposed the use of two recognition elements: recombinant forms of ZIKV NS1 and the domain III of the envelope protein (EDIII) [110], where the recognition elements were immobilized on a SPCE modified with *p*-phenylenediamine. The combination of EDIII and NS1 enables to detect *anti*-ZIKV antibodies in human serum samples without cross-reactivity to DENV by different electrochemical techniques (SWV, CV, EIS), with LOD of 57 fg/mL of monoclonal antibodies (mAb) and 17 fg/mL of mAb for EDIII and NS1, respectively. Afsahi and coworkers used PEG as an effective block against non-specific interactions at the graphene chip [113]. Biosensor chips were developed for the label-free detection of ZIKV NS1 antigen in a simulated human serum, with LOD of 4.5 × 10^−10^ mol/L. Similar works were carried out for determination of the DENV and described in chapter [68,88].

**Figure 5:**
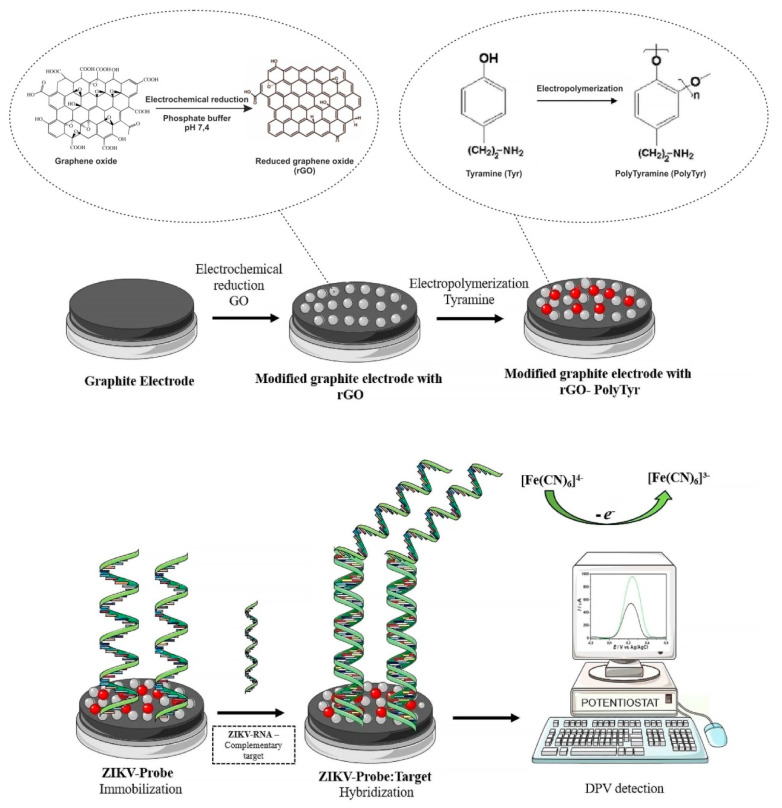
Schematic illustration of the electrochemical biosensor construction and Zika virus (ZIKV) detection. rGO—reduced graphene oxide, PTyr—polytyramine, ZIKV-Probe—ZIKV DNA probe, gRNA—genomic RNA, DPV—differential pulse voltammetry. Adapted with permission from [26].

Different materials can improve conductive properties of biosensors and thus increase their sensitivity. Various research groups reported the possibility of functionalization of electrode surfaces by polymeric materials or used them as platforms for ZIKV detection [26,48,56]. Polymeric films are also perfect materials for the conjugation with biomolecules, since they can be chemically functionalized [114]. For instance, polytyramine-conducting polymer with electrochemically reduced graphene oxide (rGO) has been used for the development of new platform for detection of genomic RNA of ZIKV in human serum samples [26]. In this work, ZIKV was quantified via DPV, with LOD down to 0.1 fg/mL, showing good stability and great potential for further practical diagnostic of ZIKV (Figure 5).

Based on the same principles, Alves and co-workers described the formation of a polymeric film derived from 3-amino-4-hydroxybenzoic acid (3-4-AHBA) on the surface of a pencil carbon graphite electrode (PCGE) for quantitative monitoring of ZIKV [56]. A proposed platform can differentiate cross-responses between DENV and ZIKV in enriched human serum. The developed material was used for the immobilization of the ssDNA ZIKV oligonucleotide and the differentiation was estimated by detection of an oligonucleotide of the genome-specific sequence of an amine-modified ZIKV. The effect of hybridization was based on current differences (Δ*I*_p_) between the probe and the complementary total ZIKV target RNA, the total RNA of DENV2, and the total RNA of DENV3. Concentration of target analyte was monitored using EIS and SWV, with LOD of 2.54 × 10^−11^ mol/L. Alternatively, Faria et al. demonstrated a platform for detection of ZIKV NS5 protein, where disposable AuEs were fabricated on polyethylene terephthalate (PET) substrate. [48]. The biosensor promised the differentiation between samples with ZIKV and DENV NS5 protein. In this system, EIS measurements showed LOD of 2.5 × 10^−8^ mol/L. Another interesting approach with polymeric materials was described by Tancharoen et al. [115]. It is based on the immobilization of surface imprinted polymers (SIPs) and GO composites at the AuE surface for detecting of ZIKV antigen. In general, SIPs have very high electrical resistance, but by adding nanocarbon materials into these SIPs an electrochemically sensitive layer is formed. In this system, CV measurements showed LOD of 2 × 10^−4^ pfu/mL in phosphate-buffered saline.

The types of electrodes, targets, and bio-recognition layers applied for the development of label-free electrochemical biosensors ZIKV described above are summarized in Table 2.

It can be concluded that authors tried to develop specific, cost-effective biosensors for ZIKV detection without cross-reaction with other flaviviruses. Present electrochemical biosensors showed practical applicability of different electrode materials for successful diagnosis of ZIKV, mostly by the EIS technique with the ferri/ferrocyanide redox probe. Commercially available ELISA tests focused on the determination of immune status ratio by dividing the optical density of the patient sample reacted with the ZIKV recombinant envelop (E) glycoprotein by the optical density of the patient sample reacted with a cross-reactive control antigen, which requires additional calculations and complicates the analysis procedure. The same holds for JEV commercial ELISA tests (Table 3).

## 5. Electrochemical Biosensors for JEV Diagnostic

JEV is endemic mostly in a large area of Asia and—as with other flaviviruses—affects the human brain membrane and causes serious damage to human health. The most significant medicine to resist the Japanese encephalitis is the use of the special vaccine. Therefore, it is important to monitor immunological products containing antigen to JEV which are used as a prevention of developing severe forms of the disease. Since most of the JEV incidents happen in countries with limited possibility to conduct diagnostic tests, it is required to develop alternative simple, user-friendly, fast, and inexpensive diagnostic tools applicable for rapid on-site analysis to eliminate the need for more expensive and labor-intensive conventional methods (ELISA, PCR) requiring more qualified personnel. Therefore, researchers have been trying to develop electrochemical biosensors as an attractive option for JEV detection in human serum or in vaccine.

In most publications, a sample containing antibodies to JEV as a bio-recognition component immobilized on the receptor layer of label-free electrochemical biosensors was investigated. For instance, Yuan and co-workers reported two studies for fast assay of JEV vaccine by the use of platinum electrode (PtE), where antiserum of JEV is used as the bio-recognition element [45,119]. In both works, bilayer of *o*-phenylenediamine (*o*-PDA) was electropolymerized on the surface of the PtE for the conjugation with antibodies to JEV. Such a modification layer has made it possible to determine JEV in vaccine amperometrically, with an LOD of 6 × 10^−9^ pfu/mL. These studies are devoted to the development of methods to control JEV vaccine [42,45,119].

**Figure 6:**
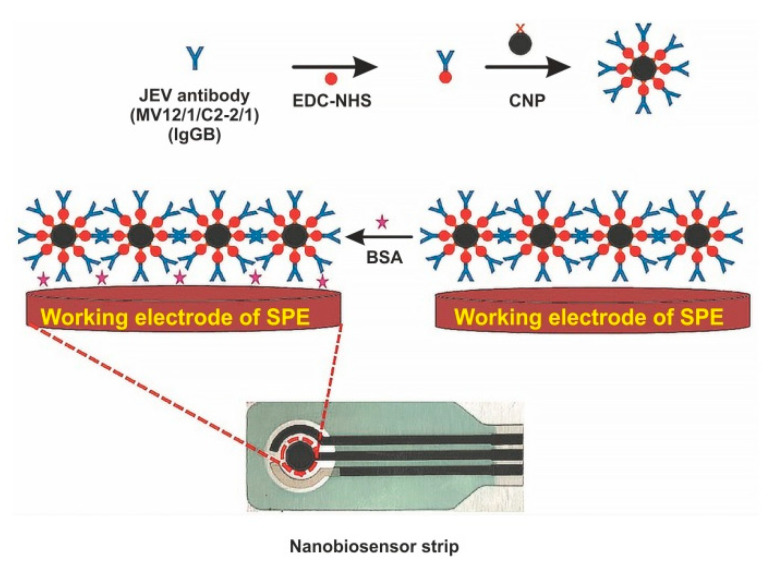
Schematic diagram of screen-printed carbon electrode (SPCE) modification for fabrication of electrochemical biosensor for the detection of Japanese encephalitis virus (JEV). EDC—1-ethyl-3-(3-dimethylaminopropyl)carbodiimide, NHS—*N*-hydroxysuccinimide, NP—carbon nanoparticle, BSA—bovine serum albumin. Adapted with permission from [46].

The immobilization of biomolecules plays an important role in the development of sensitive biosensors. Several studies paid attention to the application of nanomaterials for conjugation with a bio-recognition element. For example, Zhang et al. immobilized AuNPs and [Co(bpy)_3_]^3+^ in differently charged states on the AuE modified by l-cysteine and, afterwards, they immersed thus modified electrode into solution with the bio-recognition element (antiserum of JEV) [42]. The detection principle of JEV is based on the variation of potentiometric response before and after immunoreaction, with LOD of 3.5 × 10^−8^ pfu/mL. Another interesting approach is based on the deposition of polyaniline (PANI) nanowires at the PtE surface for immobilization of *anti*-JEV IgG [38]. The developed PANI nanowires electrochemical biosensor detects JEV by EIS, with LOD below 10 ng/mL. Carbon nanoparticles (CNPs) are also frequently exploited in JEV electrochemical biosensors. They are characterized by excellent electrochemical properties, low cost, and good deformational stability [120]. Chin and co-workers successfully applied CNPs for conjugation with JEV antibody and detection of JEV in human serum samples on a SPCE, with LOD of 2 ng/mL [121]. Another research group also modified a SPCE by CNPs for rapid and sensitive detection of JEV in human serum, with an LOD of 0.36 ng/mL [70]. Figure 6 depicts the preparation of the electrochemical biosensor for the detection of JEV with using CNPs as an immobilization platform for the JEV antibody [70].

The application of CNPs resulted in higher sensitivity in combination with EIS and CV. However, in most of these works, the possibility of cross-reactivity between flaviviruses was not discussed. Moreover, the specificity of each assay needs to be considered, because JEV is closely related to West Nile virus which frequently circulates in the same area (e.g., in India).

Table 3 summarizes types of electrodes, methods, targets, and bio-recognition layers applied for JEV determination.

It is clear that the immobilization of biomolecules with certain nanoparticles can effectively decrease LOD of JEV. EIS is most frequently used for the detection of JEV by monitoring the impedance changes of specific binding between bio-recognition element and samples containing JEV antigen. The described label-free biosensors are applicable for determination of JEV in human serum and biological products, thus paving the path for a novel method for monitoring JEV. In contrast, presented commercially available tests focused on detection of antibodies to JEV and had lower sensitivity (17–53%) in comparison with DENV and ZIKV commercial assays. In these commercial kits, false JEV IgM positive results are caused by cross-reactivity with DENV and West Nile virus.

## 6. Conclusions and Future Perspectives of Biosensors

Flaviviruses (DENV, ZIKV, and JEV) cause diseases of varying severity, from asymptomatic to the development of life-threatening haemorrhagic fever and encephalitis. Therefore, the rapid identification of flaviviruses has important clinical, economic, and epidemiological relevance. Traditional methods for diagnosing these viruses are ELISA, PCR, and PRNT, which are time-consuming, expensive, and have high requirements for trained personnel. In this sense, electrochemical biosensors become useful tools in clinical diagnosis of flaviviruses, due to the fact that determinations are fast, simple, selective, and sensitive.

The present review describes recent advances in determination of DENV, ZIKV, and JEV by label-free electrochemical biosensors (mainly voltammetric, impedimetric, and amperometric) and critically compares their possibilities and limitations in detecting flaviviruses. DNA biosensors and immunosensors measuring the electrochemical signal in the presence of redox markers, such as ferri/ferrocyanide systems and ruthenium complexes, are most frequently used. The main interest in this field is focused on increasing the selectivity of determination of flaviviruses by the modification of the biosensor’s electrode surface with natural bio-recognizing compounds (DNA and RNA, antigens, antibodies). In this field, more reliable results were obtained by using non-structural protein (NS1), which is a clinical indicator in immunosensing and molecular research for diagnostic of flaviviruses, as a bio-recognition element or an object for assays in various samples. In addition to natural bio-molecules, their synthetic analogues—synthetic proteins (polymers with molecular fingerprints that are imitators of antibodies or antigens) are used to reduce the costs of analysis. Necessary density and binding efficiency of bio-recognition elements for sensitive determination of flaviviruses was achieved by various modifications of electrode surface with graphene oxide, metal NPs, metal oxides, carbon nanotubes, polymer films, and conductive MIPs. In particular, the polymer films have a great advantage, because they allow receptor molecules to maintain their native conformation and binding activity.

The major challenge in this area is the development of a specific biosensor for differential diagnostics of flaviviruses without cross-reactivity with viruses that have similar genomes and similar antigenic structure. This factor could limit the application of biosensors, and, thus, considerable research is still necessary in this field. Fortunately, much has been learned over the past decade about flaviviruses, and investigators suggested some optimizations followed by successful results for the identification of the specific virus serotype. However, in many papers, the proposed electrochemical label-free biosensors are fabricated by relatively labor-intensive ways, without information about the stability, storage conditions, and shelf-life of the biosensors. So far, no label-free sensors for tick-borne encephalitis virus were introduced, so that research in this field could be interesting.

Despite still existing limitations in the widespread usage of label-free electrochemical biosensors for the detection of flaviviruses in medical diagnostics, their potential with high sensitivity, low consumption of analyzed objects, low matrix influence, relatively low investment and running costs, and suitability for miniaturization has not only reached, but exceeded the potential of other analytical methods available for these purposes.

The most prospective pathways for future research in the field of flaviviruses sensors is probably the label-free approach based on novel nanomaterials, which is a prevailing trend in most fields of electroanalytical chemistry. However, further effort should be given to introducing novel method into practical laboratories, and we hope that this review can be a small contribution in this important effort.

## Figures and Tables

**Table 1:** Survey of electrochemical label-free biosensors and commercially available assays for dengue diagnostic.

**Electrochemical Label-Free Biosensors**								
**Electrode/Platform ** **Material**	**Method**	**Bio-Recognition Layer**	**Target**	**Limit of Detection**	**Possible Cross-Reactivity**	**Adv.**	**Dis.**	**Ref.**
A nanoporous alumina membrane/Pt	DPV, CV	Mouse anti-DENV2 monoclonal antibody	DENV2	1 pfu/mL	Chikungunya virus, West Nile virus, DENV3	a, f	b, d, e	[87]
A nanoporous alumina membrane/Pt	DPV, CV	DENV probe *ss*DNA	DENV1	9.55 × 10^−12^ mol/L	DENV3	e, f	b, d, e	[81]
Mn_2_O_3_/GCE	DPV	DENV probe *ss*DNA	DENV comple- mentary DNA	1.2 × 10^−19^ mol/L	DENV non-complementary DNA	d, e, f	c, e	[81]
Nafion/ITO	SWV	DENV probe *ss*DNA	DENV2 RNA	2 × 10^−18^ mol/L	RNAs (DENV1, −3, −4)	a, e	b, e, f	[66]
Au nanostructures/Ti	DPV, CV	DENV thiolated probe *ss*DNA	DENV comple- mentary DNA	9.7 × 10^−16^ mol/L	DENV non-comple- mentary DNA, human serum albumin	b, c, e	e	[80]
AuE	SWV, EIS	Synthetic peptides (DGV BP1–BP5)	DENV NS1antigen	1.49 μg/mL	Bovine serum albumin	a, f	b, e	[68]
MUA/6COH/AuE	EIS	Anti-DENV NS1 antibody	DENV NS1 antigen	30 ng/mL	–	b, e, f,	c, e	[37]
A nanoporous alumina electrode	EIS	Anti-DENV2 antibody	DENV2	1 pfu/mL	Chikungunya virus, West Nile virus	a, f	b, d, e	[93]
Pt film/alumina membrane	EIS	Anti-DENV2 antibody	DENV2 DENV3	0.23 and0.71 pfu/mL	Chikungunya virus	a, b, e	c, d, e, f	[57]
4-mercaptobenzoic acid/AuNPs/AuE	EIS	Anti-DENV antibody	DENV1–4	–	–	a, b,	c, e	[99]
AuNPs/1,4-phenylenediamine/ITO	EIS	Anti-DENV NS1 antibody	DENV NS1antigen	5 ng/mL	Malaria-infected sera	b, d, e, f	a, c, f	[88]
1-pyrenebutyric acid/SWNT/Au microelectrode	EIS	Anti-DENV NS1 antibody	DENV NS1 antigen	1 ng/mL	Artificial human saliva	a, b, e	b, c, d, e	[89]
11-(ferrocenyl)undecanethiol/PEG (poly(ethylene glycol)- thiol/AuE	EIS	Anti-DENV NS1 antibody, DENV NS1 antigen	DENV NS1 antigen, *anti-*DENV NS1 antibody	1.2 ng/mL, 6.1 ng/mL	–	b, d, e	a, c, d, e, f	[50]
Ferrocene-tagged peptide/AuE	EIS	Anti-DENV NS1 antibody	DENV NS1 antigen	–	–	b, d	c, d, e, f	[90]
Poly(4-aminobenzoic acid)/screen-printed electrode	EIS	DENV NS1 antigen	*Anti-*DENV NS1 antibody	–	Uric acid, glucose, water, HBS-EP buffer	b, c, d, f	c	[91]
Copolymers + graphene oxide/AuE	EIS	DENV antigen	DENV2 antibody	0.12 pfu/mL	Influenza A virus	b, e	c, d, e, f	[100]
Dopamine/polysulfone nanofibers/SPCE	EIS	Imprinted NS1 protein	DENV NS1 antigen	0.3 ng/mL	Fetal bovine serum, lysozyme	b, c, d, e, f	c, d, e	[95]
PVB (polyvinyl formal chloroform solution)—Fe_3_O_4_/AuE	EIS, CV	CramoLL (lectin and fetuin isolated from Cratylia mollis seeds)	Glycoproteins of DENV2, DENV3	–	–	f	b, c, d, e	[73]
PNA/SiNW (silicon nanowire)	Electronic conductivity	DENV comple- mentary fragment	DENV2	1.0 × 10^−14^ mol/L	–	b, c, f	b, c, d, e	[86]
AuE	Electronic conductivity	Anti-DENV NS1 antibody	DENV NS1antigen	0.25 μg/mL	–			[98]
Anion exchange nanoporous membrane	Conducto-metry	Negatively charged DNA oligoprobes	DENV2, DENV3 RNA	1.0 × 10^−12^ mol/L	–	b, d, f	e	[44]
**Commercially Available Assays**								
**Platform/Com-pany**	**Method**	**Bio-Recognition Molecules**	**Target**	**Detection Rates**	**Possible Cross-Reactivity**	**Adv.**	**Dis.**	**Ref.**
Test Strips/Abbott SD BIOLINE Dengue Duo	*In-vitro* immunochromatogra-phic	DENV envelope proteins—Au colloidal	DENV NS1 antigen, DENV Ig M, Ig G Antibodies	92.4%	RNAs (DENV1, −3, −4) and other flaviviruses	b, d, e, f	a, c	[101,102]
Microtiter plate/Panbio Dengue Ig M	Membrane attack complex—ELISA	Anti-DENV human-IgM antibody, DENV NS1 antigen, antibody-HRP conjugates	DENV Ig M antibodies	81%	RNAs (DENV1, −3, −4) and other flaviviruses	d, f	a, c	[103,104]
Microtiter plate/Abbott SD ELISA Dengue	Indirect ELISA	Anti-DENV human-IgM antibody, DENV NS1antigen, antibody-HRP conjugates	DENV Ig M antibodies	69.2%	RNAs (DENV1, −3, −4) and other flaviviruses	d, f	a, c	[103,105]

**Advantages: a**—lower cost of analysis; **b**—rapid response time; **c**—portability; **d**—opportunity to quantify biomolecules in biological liquids; **e**—high sensitivity; **f**—representative information about reproducibility and stability. **Disadvantages: a**—higher cost of analysis; **b**—suitable only for primary screening and require confirmation of positive results by independent methods; **c**—complex electrode/platform fabrication; **d**—limited or unknown sensor stability; **e**—limited or unknown shelf-life of the sensor; **f**—limited sensor reproducibility.

**Table 2:** Survey of electrochemical label-free biosensors for Zika diagnostic.

**Electrode** **Material**	**Method**	**Bio-Recognition Layer**	**Target**	**Limit of Detection**	**Possible Cross-Reactivity**	**Adv.**	**Dis.**	**Ref.**
DTSP (dithiobis(succi-nimidyl propionate))/IDE (interdigitated micro-Au electrode)	EIS	ZIKV envelope protein antibody (Zev-Abs)	ZIKV antigen	1 × 10^−11^ mol/L	Chikungunya virus, West Nile virus, DENV	c, e, f	b, c	[49]
*p*-Phenylenediamine/SPCE	EIS, CV, SWV	ZIKV EDIII and NS1	ZIKV antibodies	17 fg/mL	DENV	d, e	a, c	[110]
PEG/Ti-Pt leads on SiO_2_/graphene chip	Capacitan-ce measure-ment	Mouse *anti*- ZIKV monoclonal antibody	ZIKV NS1 antigen	4.5 × 10^−10^ mol/L	JEV	b, c	a, c, e	[113]
SIPs-GO composites/AuE	DPV	ZIKV imprinted to the polymer	ZIKV antigen	2 × 10^−4^ pfu/mL	DENV2	d	a, c, d	[115]
3-4-AHBA/ PCGE	SWV, EIS	ZIKV aminated ssDNA	ZIKV antigen	2.54 × 10^−11^ mol/L	DENV2, −3	f	b, c, e	[56]
ZnO nanostructures/PCB	CV	*Anti-*ZIKV NS1 antibody	ZIKV NS1 antigen	1 pg/mL	DENV NS1 antigen	e, f	a, c, e	[109]
Disposable AuE/PET	EIS, DPV, CV	ZIKV thiolated probe *ss*DNA	ZIKV NS5 antigen	2.5 × 10^−8^ mol/L	DENV NS5 protein	a, c	b, e	[48]
Poly-tyramine/ rGO/graphite electrode	DPV	ZIKV oligonucleotide	ZIKV genomic RNA	0.1 fg/mL	–	d, f	f	[26]
**Commercially Available Assays**								
**Platform/Company**	**Method**	**Bio-Recognition Molecules**	**Target**	**Detection Rates**	**Possible Cross-Reactivity**	**Adv.**	**Dis.**	**Ref.**
Test Strips/Abbott SD BIOLINE Zika Ig M	*In-vitro* immunochromatographic	ZIKV envelope proteins—Aucolloidal	ZIKV NS1 antigen, DENV Ig M, Ig G antibodies	95.6%	RNAs (DENV1, −3, −4) and other flaviviruses	b, d, e, f	a, c	[101]
Microtiter plate/InBios ZIKV Detect™	Membrane attack complex—ELISA	*Anti-*DENV human-IgM antibody, DENV NS1antigen, antibody-HRP conjugates	DENV Ig M antibodies	96.5%	Yellow Fever virus, Chikungunya virus	d, e, f	a, c	[116,117]
Microtiter plate precoated with ZIKV NS1/ Euroimmun anti-ZIKV IgM	Indirect ELISA	Antibody-HRP conjugates	DENV Ig M antibodies	56%	West Nile virus	d, f	a, c	[118,117]

**Advantages: a—**lower consumption of chemical reagents; **b—**rapid response time; **c—**portability; **d—**opportunity to quantify biomolecules in human serum; **e—**high sensitivity; **f—**representative information about reproducibility and stability. **Disadvantages: a**—higher consumption of chemical reagents; **b**—suitable only for primary screening and require confirmation of positive results by independent methods; **c**—complex electrode/platform fabrication; **d—**limited or unknown sensor stability; **e**—limited or unknown shelf-life of the sensor; **f**—limited sensor reproducibility.

**Table 3:** Survey of electrochemical label-free biosensors for Japanese encephalitis diagnostic.

**Electrode** **Material**	**Method**	**Bio-Recognition Layer**	**Target**	**Limit of Detection**	**Adv.**	**Dis.**	**Ref.**
Nano-Au/*o-*PDA polymer film/PtE	Amperometry	Antiserum of JEV	JEV antigen	6 × 10^−9^ pfu/mL	b, f	c, b	[119]
Nano-Au/*o-*PDA polymer film with deposited Prussian blue/PtE	Amperometry	Antiserum of JEV	JEV antigen	6 × 10^−9^ pfu/mL	b, f	a, b, c	[45]
l-cysteine + nano-Au and [Co(bpy)_3_]^3+^/AuE	Potentiometry	Antiserum of JEV	JEV antigen	3.5 × 10^−8^ pfu/mL	b	a, b, c	[42]
Silanized surface with protein A/screen-printed electrode	EIS	Serum containing antibodies to JEV	JEV antigen	0.75 µg/mL	a, b c, d	d, e, f	[122]
PANI nanowires/PtE	EIS	*Anti*-JEV antibodies	JEV antigen	10 ng/mL	e	c, d, e, f,	[38]
CNPs/3-aminopropyl triethoxysilane/SPCE	EIS, CV	JEV antibody	JEV antigen	2 ng/mL	b, d	c, d, e, f,	[121]
PANI/multiwalled carbon nanotubes/PtE	EIS	*Anti*-JEV antibodies	JEV antigen	–	a, b	d, e, f	[27]
CNPs/chitosan/SPCE	EIS, CV	JEV antibody	JEV antigen	0.36 ng/mL	b, d, c	a, c, d, e, f	[70]
**Commercially Available Assays**							
**Platform/Company**	**Method**	**Bio-Recognition Molecules**	**Target**	**Detection Rates**	**Adv.**	**Adv.**	**Ref.**
Microtiter plate/InBios JEV Detect™	Membrane attack complex—ELISA	*Anti-*JEV human-IgM antibody, JEV NS1 antigen, antibody-HRP conjugates	JEV Ig M antibodies	56%	d, f	a, c	[123,124]
Microtiter plate/XCyton JEV Chex	Membrane attack complex—ELISA	*Anti-*JEV human-IgM antibody, JEV NS1 antigen, antibody-HRP conjugates	JEV Ig M antibodies	19%	d, f	a, c	[124]

**Advantages: a—**lower consumption of chemical reagents; **b—**rapid response time; **c—**portability; **d—**opportunity to quantify biomolecules in human serum; **e—**high sensitivity; **f—**representative information about reproducibility and stability. **Disadvantages: a**—higher consumption of chemical reagents; **b**—suitable only for primary screening and require confirmation of positive results by independent methods; **c**—complex electrode/platform fabrication; **d—**limited or unknown sensor stability; **e**—limited or unknown shelf-life of the sensor; **f**—limited sensor reproducibility.
